# Efficient Ethylene/Ethane Separation Achieved by a Temperature-Responsive Flexible MOF

**DOI:** 10.3390/molecules31162875

**Published:** 2026-08-18

**Authors:** Sheng Liu, Xuefu Tian, Yutong Zou, Yanxi Li, Yawen Bo, Chuanqi Wu, Kebin Chi, Dejun Shi, Qihan Gong

**Affiliations:** Fundamental Science & Advanced Technology Lab, PetroChina Petrochemical Research Institute, Beijing 102200, China; liusheng025@petrochina.com.cn (S.L.); fuhua1751285711@163.com (X.T.); zouyutong010@petrochina.com.cn (Y.Z.); liyanxi@petrochina.com.cn (Y.L.); boyawen@petrochina.com.cn (Y.B.); 13903105514@163.com (C.W.); ckb459@petrochina.com.cn (K.C.); shidejun010@petrochina.com.cn (D.S.)

**Keywords:** metal–organic framework, ethylene/ethane separation, flexibility

## Abstract

This study investigates the temperature-responsive thermodynamic behavior of a zinc-based flexible metal–organic framework for ethylene/ethane C_2_H_4_/C_2_H_6_ separation. Isothermal adsorption suggested a highly sensitive, temperature-dependent guest-induced structural transition. At 273 K, C_2_H_4_ triggers a pronounced gate-opening effect at low pressures with a broad desorption hysteresis loop, while C_2_H_6_ shows only a marginal stepwise uptake increase at about 0.6 bar. Crucially, elevating the temperature to 298 K shifts the C_2_H_4_ transition threshold to higher pressures and completely suppresses the C_2_H_6_ response. This divergence enables the selective triggering of the framework expansion exclusively by C_2_H_4_ at room temperature. Further heating to 323 K entirely suppresses phase-transition characteristics for both gases. Dynamic mixed-gas breakthrough experiments confirm that precise thermal regulation strikes an optimal balance, achieving maximized fixed-bed separation selectivity while preserving a substantial capacity. This work demonstrates temperature as a key parameter to tailor the dynamic separation performance of flexible adsorbents in practical PSA/TSA applications.

## 1. Introduction

The separation of ethylene/ethane (C_2_H_4_/C_2_H_6_) mixtures remains a long-standing challenge in the petrochemical industry [1,2,3,4,5]. Adsorptive separation utilizing porous solid materials has emerged as a highly promising, energy-efficient alternative to conventional cryogenic distillation, enabling efficient C_2_H_4_ purification under mild operating conditions. In contrast, traditional cryogenic distillation demands harsh environments (183–258 K and 7–28 bar) and complex columns requiring over 100 theoretical trays, resulting in an immense energy consumption of up to 7.3 GJ t^−1^ [6,7]. To mitigate these drawbacks, various adsorbents—including zeolites [8,9], porous carbons [10,11], and porous coordination polymers (PCPs) [12,13]—have been extensively explored. Among these, metal–organic frameworks (MOFs), a distinct class of crystalline PCPs, stand out as premier candidates for C_2_H_4_/C_2_H_6_ separation [14,15,16,17,18]. This is primarily attributed to their precisely tunable pore apertures, tailorable chemical microenvironments, and exceptional structural flexibility, which facilitate the targeted optimization of both adsorption capacity and selectivity via functionalization strategies [19,20,21,22,23].

For ethylene-preferential adsorption, selective and strong binding can be achieved by incorporating open metal sites (OMSs) [13,16,24] or engineering π-complexation interactions [25,26] within the framework. Additionally, precise size-sieving mechanisms [14,15] or pore-diffusion kinetic effects [27] can be exploited to physically separate the two molecules or induce significant disparities in their diffusion rates. Furthermore, to minimize the energy penalty associated with desorption and directly harvest high-purity ethylene, reverse separation strategies targeting preferential ethane adsorption have garnered significant attention [6]. Innovative approaches such as introducing electronegative atoms into pore walls to establish multiple hydrogen bonds [6,28], designing specific binding sites (e.g., peroxo sites [17]) to amplify van der Waals forces [18], and harnessing the “gate-opening” effects of flexible MOFs for phase-transition-driven separation [29] offer promising avenues for overcoming current separation bottlenecks.

Flexible MOFs exhibit unique advantages in gas separation due to their temperature- and pressure-dependent separation capabilities, which arise from dynamic structural responses such as gate-opening effects and reversible framework transformations [12,30,31,32,33,34,35,36,37,38]. Consequently, numerous studies have explored their flexible behavior for adaptive C_2_H_4_/C_2_H_6_ separation. For instance, IITKGP-39 [39], TYUT-17 [29] and MAF-42 [40] demonstrate gate-opening behavior in response to C_2_H_4_ and C_2_H_6_ adsorption, with the gate-opening pressure showing a positive correlation with temperature. Especially the transition pressures of MAF-42 are strongly temperature-dependent, showing a significant increase as the temperature rises from 273 K to 298 K and 323 K, with values of 10.6/4.2, 16.6/7.7, and 25.4/11.4 atm for C_2_H_4_ and C_2_H_6_, respectively. At 298 K, C_2_H_6_ is completely excluded below the pore-opening pressure of 7.7 atm, giving single component selectivity of ca. 300. In X-dia-1-Ni series [36] of MOFs, C_2_H_6_ can induce a structural transformation from the narrow-pore (NP) phase to the large-pore (LP) phase at 263 K and 273 K under specific pressures, whereas C_2_H_4_ cannot induce this phase transition. While at 298 K, neither ethane nor ethylene can trigger the phase transition. The LP phase of X-dia-1-Ni_0.89_Co_0.11_ exhibited a high uptake of C_2_H_6_ (111 cm^3^/g, 1 atm, 273 K) and a low uptake of C_2_H_4_ (12.2 cm^3^/g).

**Scheme 1:**
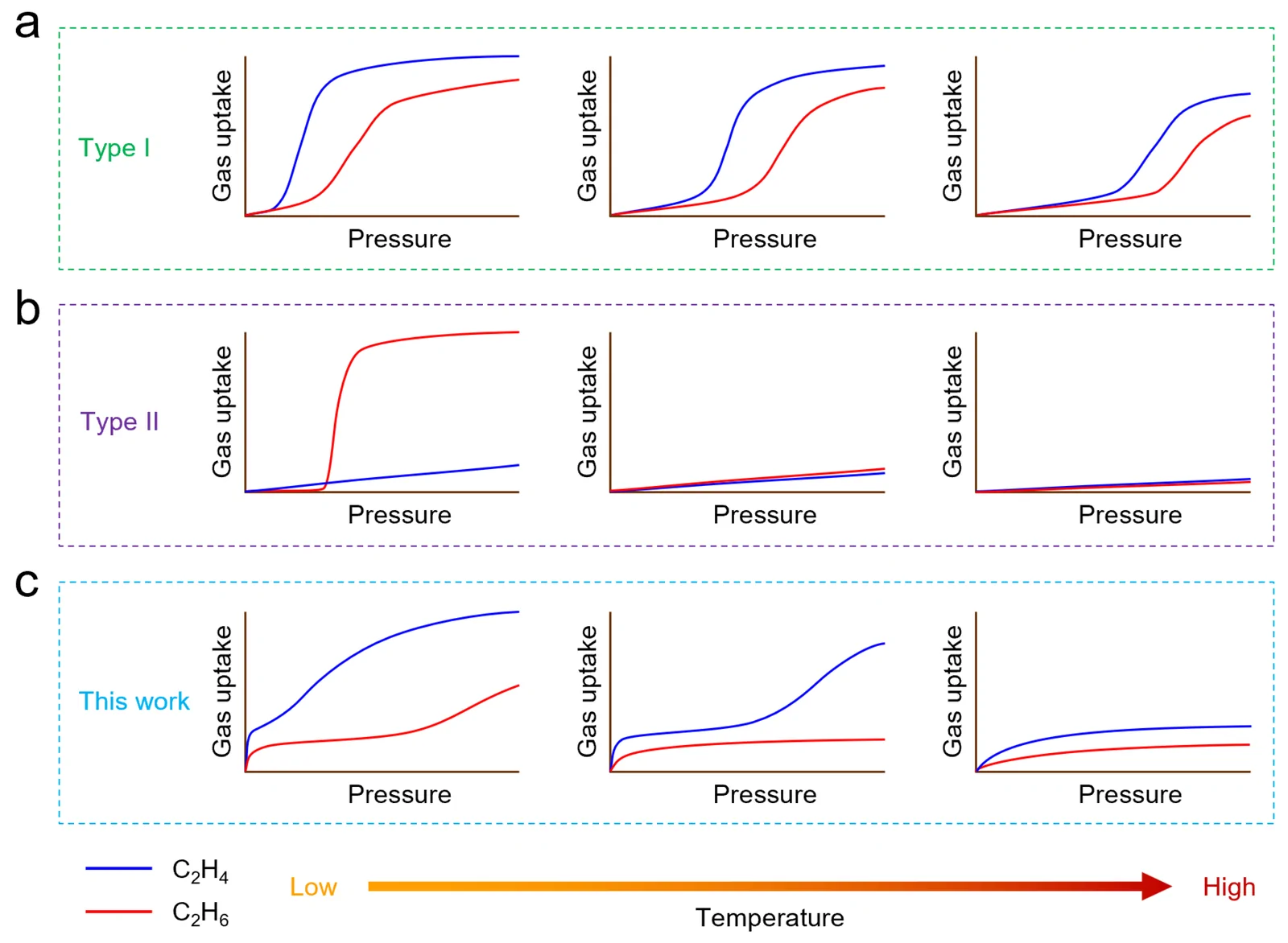
Temperature-driven divergent responsiveness of flexible MOFs, (**a**) Type I; (**b**) Type II; (**c**) This work.

Herein, we present a unique temperature-dependent guest-induced adsorption behavior on ZnPaDa, a flexible MOF constructed from 1,2,4,5-benzenetetracarboxylic acid (pyromellitic acid, Pa) and 3,5-diamino-1,2,4-triazole (Da). As shown in Scheme 1, unlike conventional flexible adsorbents that either exhibit a parallel thermal shift in gate-opening pressure for both gases (Scheme 1a), or inherently lack responsiveness for one specific guest (Scheme 1b), ZnPaDa demonstrates a hierarchical, temperature-driven divergent transition (Scheme 1c). It undergoes a unique cascade evolution: from a dual-gas responsive state at lower temperature, resolving into a highly selective single-gas gate-opening state at moderate temperature, and ultimately transitioning into a completely non-responsive state for both gases at higher temperature. The distinct variation behaviors of C_2_H_6_ and C_2_H_4_ adsorption across the tested range identify 298 K as the optimal point for C_2_H_4_/C_2_H_6_ separation, where ZnPaDa exhibits the highest uptake ratio while maintaining a favorable C_2_H_4_ capacity.

## 2. Results and Discussion

### 2.1. Crystal Structure of ZnPaDa

**Figure 1:**
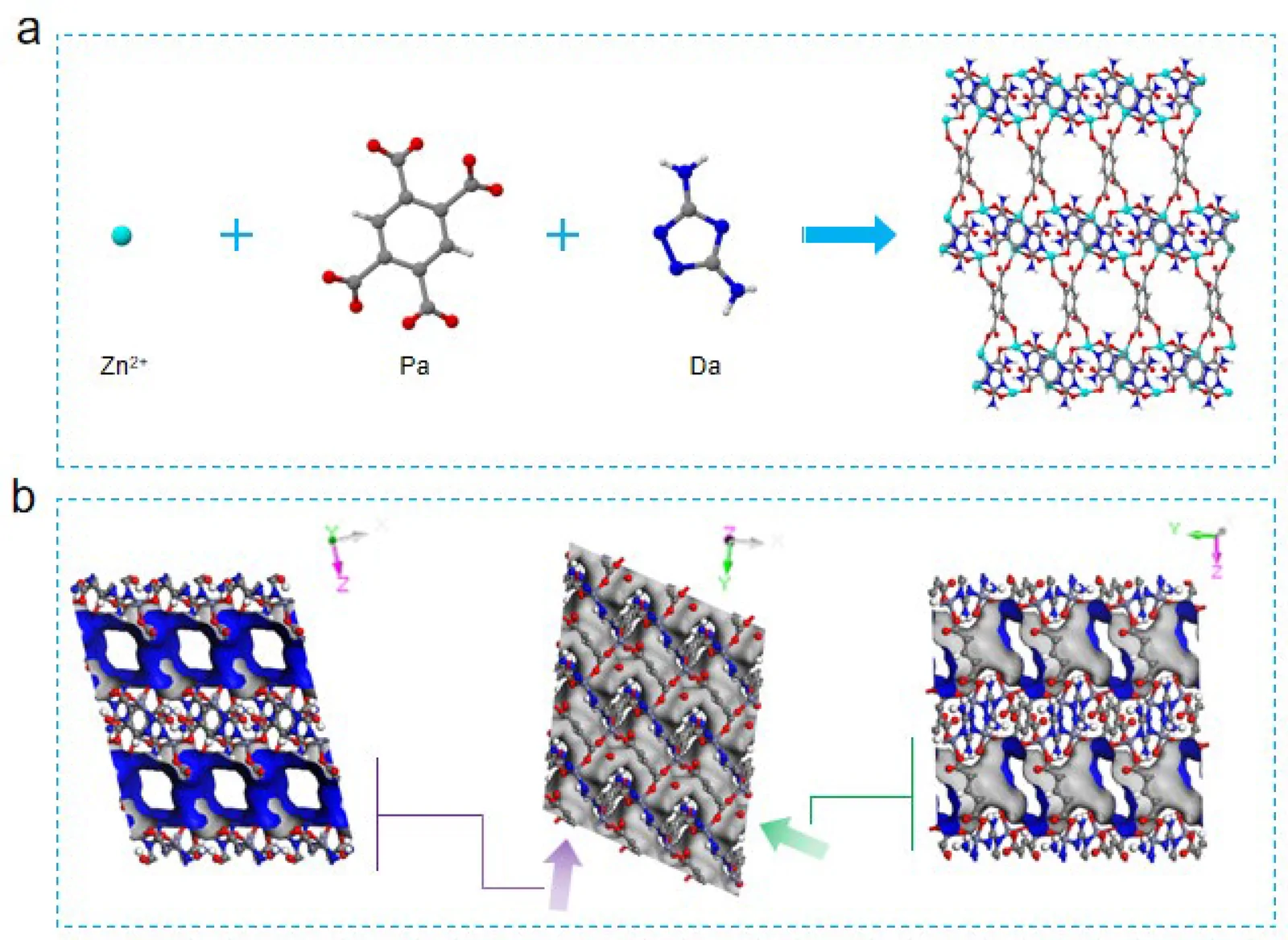
Crystal framework (**a**) and pore structure (**b**) of ZnPaDa.

The crystal structure of ZnPaDa [41] is constructed from Zn(II) ions, pyromellitate ligands (btec^4−^), and protonated 3,5-diamino-1,2,4-triazole ligands (Hdatrz), giving rise to a three-dimensional coordination network, as shown in Figure 1a. The asymmetric unit contains two crystallographically independent Zn(II) centers, Zn1 and Zn2, both of which adopt distorted tetrahedral coordination geometries. The Zn1 center is coordinated by two carboxylate oxygen atoms from two different btec^4−^ ligands and two triazole nitrogen atoms from two different Hdatrz ligands, forming a ZnO_2_N_2_ coordination sphere. In contrast, the Zn2 center is surrounded by three carboxylate oxygen atoms from three different btec^4−^ ligands and one triazole nitrogen atom from one Hdatrz ligand, resulting in a ZnO_3_N tetrahedral environment. The carboxylate groups of the btec^4−^ ligands display diverse coordination modes and connect adjacent Zn(II) centers through Zn–O bonds, extending the inorganic nodes in multiple spatial directions and constructing the primary skeleton of the structure. Meanwhile, the Hdatrz ligands adopt a μ-1,2,4-bridging coordination mode, in which the triazole nitrogen atoms link different Zn(II) ions, contributing to the formation of Zn_2_ dinuclear units and stabilizing the three-dimensional network. Through the synergistic coordination of btec^4−^ and Hdatrz ligands, the Zn(II) nodes are further extended into a three-dimensional mixed-ligand coordination framework. Within the architecture, btec^4−^ acts as the primary structural linker and pillar ligand, whereas Hdatrz serves as an N-donor bridge that modulates the connectivity of metal nodes and the overall topology of the network.

ZnPaDa contains interconnected two-dimensional channels (Figure 1b). The center image provides a view of the channels from the [001] direction, where the channels along the purple and green arrows can be seen intersecting and connecting to form two-dimensional channels. The pore aperture is square when viewed along the [010] direction, and the amino groups are distributed on the inner wall of the channel. While the pore aperture is elongated when viewed approximately along the [100] direction.

### 2.2. Characterization of ZnPaDa Samples

**Figure 2:**
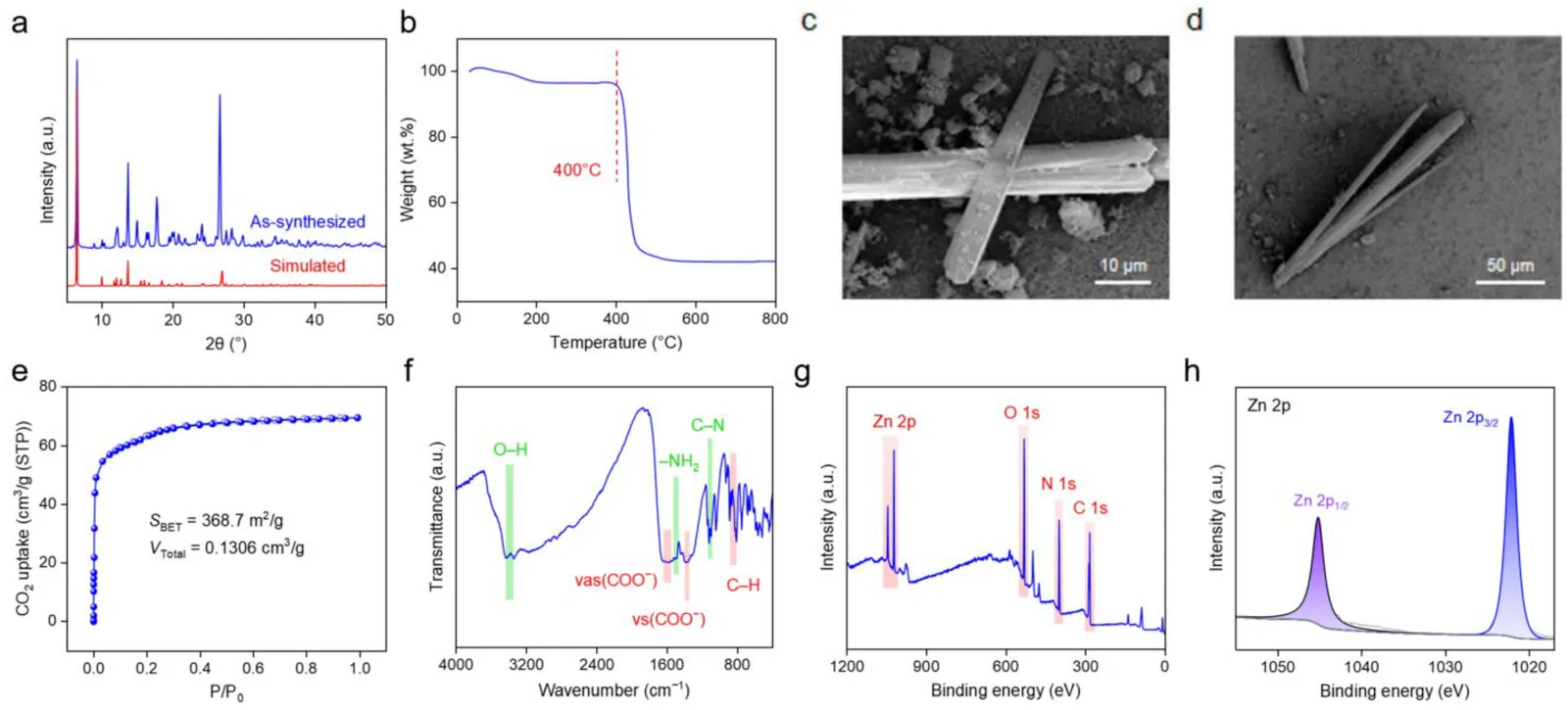
Characterization of ZnPaDa samples. (**a**) Powder X-ray diffraction (PXRD) pattern of the as-synthesized ZnPaDa; (**b**) Thermogravimetric analysis (TGA) curve of ZnPaDa; (**c**,**d**) Scanning electron microscopy (SEM) images of ZnPaDa at magnifications of (**c**) 2000× and (**d**) 500×; (**e**) CO_2_ adsorption isotherm at 195 K of ZnPaDa; (**f**) FTIR spectrum of ZnPaDa. (**g**) XPS survey spectrum of ZnPaDa. (**h**) High-resolution XPS spectrum of the Zn 2p region for ZnPaDa.

Powder X-ray diffraction (PXRD) was employed to characterize the phase purity and crystallinity of the ZnPaDa sample. As shown in Figure 2a, the PXRD pattern of the synthesized ZnPaDa sample matches well with that simulated from the single-crystal structure data, with a high-intensity and sharp primary characteristic diffraction peak observed at 2θ = 6.5° and no obvious impurity peaks, indicating the successful preparation of ZnPaDa crystals with high purity and high crystallinity.

To investigate the thermal stability of the ZnPaDa, a thermogravimetric analysis was conducted, and the resulting thermogravimetric curve is shown in Figure 2b. The sample mass remained essentially constant within the temperature range of 60–400 °C, indicating that ZnPaDa possesses excellent thermal stability. When the temperature rose to approximately 400 °C, the sample exhibited significant and rapid weight loss, a phenomenon attributed to the thermal decomposition of the organic ligands within the material’s framework, which subsequently led to the collapse of the framework structure and the eventual complete calcination of the sample into the corresponding metal oxide. The thermogravimetric analysis results indicate that the framework structure of ZnPaDa is stable up to at least 400 °C. Even after the guest molecules are completely removed, its framework structure can still maintain good stability and crystallinity.

Scanning electron microscopy (SEM) images (Figure 2c,d) reveal that ZnPaDa primarily consists of one-dimensional (1D) rod- or needle-like microcrystals with high aspect ratios. These crystals exhibit well-defined outlines with regular, sharp edges, with some individuals intersecting or stacking into bundles. Their surfaces are relatively smooth, displaying minor nanoparticle attachment, alongside a small number of coexisting irregular blocky fragments in the background. The rod-like structures can extend up to several tens of micrometers in length and feature distinct rectangular or polygonal cross-sections, underscoring their excellent overall crystallinity.

As shown in Figure 2e, the CO_2_ adsorption isotherm of ZnPaDa displays a sharp uptake in the ultra-low pressure region (P/P_0_ < 0.1) followed by a distinct plateau at higher pressures. This reversible, classic Type I isotherm profile confirms the strictly microporous nature of the framework, where micropore filling serves as the primary adsorption mechanism. The steep initial slope reflects an excellent size-matching and strong affinity between the CO_2_ probes and the host channels, facilitating a rapid pore-filling process. Furthermore, Horvath–Kawazoe (HK) analysis reveals that the pore size is predominantly centered at 5.9 Å (Figure S1), complementary to a Brunauer–Emmett–Teller (BET) specific surface area of approximately 368.7 m^2^/g.

Fourier transform infrared (FTIR) spectroscopy was employed to analyze the coordination structure of ZnPaDa, as illustrated in Figure 2f. The spectrum displays the characteristic absorption bands originating from both pyromellitic acid and 3,5-diamino-1,2,4-triazole ligands. Specifically, the broad band centered near 3400 cm^−1^ is assigned to the O-H stretching vibration (arising from moisture adsorbed during KBr pellet preparation) overlapping with the N-H stretching of the triazole amino groups. The weak peak near 3100 cm^−1^ corresponds to the aromatic C-H stretching of the benzene ring. In the mid-IR region, the prominent absorption near 1620 cm^−1^ represents the asymmetric stretching vibration of the carboxylate groups (COO^−^). This is accompanied by a bending vibration of -NH_2_ and triazole ring skeletal vibrations near 1550 cm^−1^, followed by the symmetric COO^−^ stretching vibration at approximately 1420 cm^−1^. Notably, the distinct peak observed near 1100 cm^−1^ is attributed to the C-N stretching vibration within the triazole ring. At lower wavenumbers, the out-of-plane C-H bending of the benzene ring appears near 800 cm^−1^. Furthermore, successful metal-ligand coordination is confirmed by the emergence of characteristic framework vibrations in the fingerprint region, where the peaks near 700 cm^−1^ and 500 cm^−1^ are unambiguously assigned to the Zn-O and Zn-N stretching modes, respectively.

X-ray photoelectron spectroscopy (XPS) was employed to investigate the surface elemental composition and chemical valence states of ZnPaDa. As illustrated in the survey spectrum (Figure 2g), the characteristic peaks of O 1 s (~530 eV), N 1 s (~400 eV), and C 1 s (~285 eV) are clearly resolved at lower binding energies, alongside the Zn 2p signal, confirming the successful incorporation of zinc and the presence of framework-constituting elements. To further elucidate the coordination state of zinc, the high-resolution Zn 2p spectrum was examined (Figure 2h). The spectrum splits into a well-defined spin–orbit doublet, consisting of a Zn 2P_3/2_ peak at 1021 eV and a Zn 2P_1/2_ peak at 1045 eV. The resulting energy separation of approximately 24 eV is in excellent agreement with standard reference data for Zn^2+^. Moreover, the absence of any multi-valence features or noticeable satellite peaks demonstrates that zinc exists stably in a single +2 oxidation state, consistent with its typical coordination behavior in such crystalline frameworks.

### 2.3. Discussion on Single-Component Adsorption Behavior

**Figure 3:**
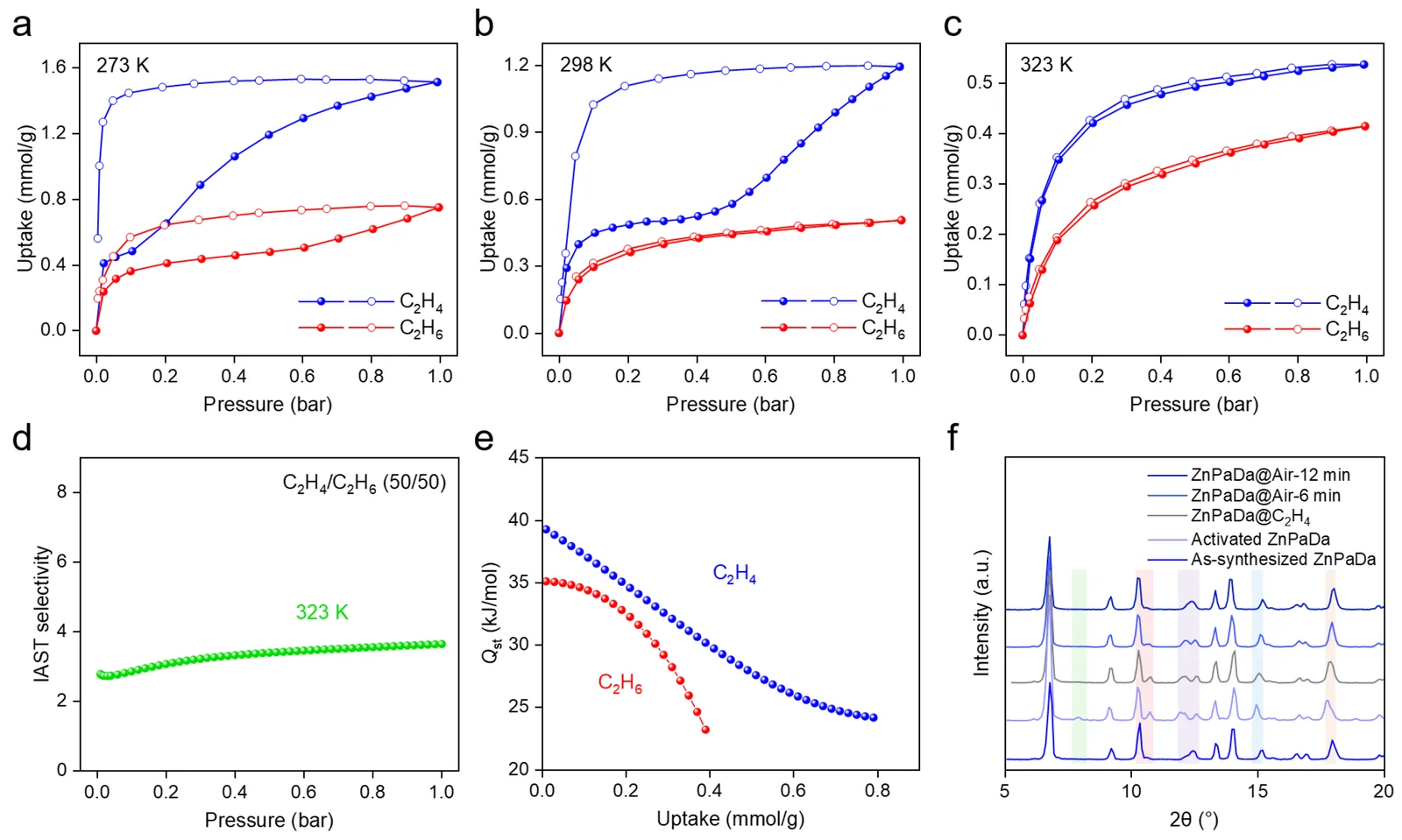
Single component adsorption experiments of ZnPaDa samples at 273 K (**a**), 298 K (**b**) and 323 K (**c**); (**d**) IAST selectivity of ZnPaDa under C_2_H_4_/C_2_H_6_ (50/50, *v*/*v*) mixture; (**e**) Q_st_ of C_2_H_4_/C_2_H_6_; (**f**) Powder X-ray diffraction (PXRD) patterns of as-synthesized, activated, C_2_H_4_ adsorbed, activated sample upon air exposure for 6 min, activated sample upon air exposure for 12 min of ZnPaDa.

The single-component adsorption isotherms of C_2_H_4_ and C_2_H_6_ on the ZnPaDa material at 273 K, 298 K, and 323 K are shown in Figure 3a–c.

As can be seen from Figure 3a, at 273 K the framework exhibits a differential response behavior towards C_2_H_4_ and C_2_H_6_. In the adsorption branch of C_2_H_4_, the framework initiates a progressive flexible phase transition at a remarkably low pressure (gate-opening pressure ~0.05 bar). The adsorption uptake increases continuously with pressure, reaching a fully open state at 1.0 bar with a capacity of approximately 1.5 mmol/g. Subsequently, in the desorption branch of C_2_H_4_, the isotherm exhibits an exceptionally broad desorption hysteresis loop. During the depressurization process, the adsorption capacity is locked at a high level over a wide pressure range, and a sharp, cliff-like desorption only occurs when the system pressure drops to an extremely low level (<0.05 bar). In contrast, in the adsorption branch of C_2_H_6_, the van der Waals forces of C_2_H_6_ are insufficient to overcome the framework deformation energy in the low-pressure region, restricting its initial adsorption primarily to the intrinsic pores. However, as the pressure increases to approximately 0.6 bar, a slight increase in the slope of the isotherm emerges, suggesting that C_2_H_6_ is only then able to induce a weak localized gate-opening or secondary phase transition, culminating in a total uptake of merely 0.75 mmol/g at 1.0 bar. In the desorption branch of C_2_H_6_, only a moderately narrow hysteresis loop is observed, indicating that the guest molecules can more easily escape as the pressure decreases.

The adsorption behavior of the material at 298 K for the two gases (Figure 3b) is distinct, with C_2_H_4_ continuing to show gate-opening effect while C_2_H_6_ exhibits a typical type I isotherm. In the adsorption branch of C_2_H_4_, the isotherm exhibits a typical “S”-shaped step profile. In the low-pressure region, the adsorption increases rapidly and gradually approaches a plateau; however, once the pressure reaches the gate-opening threshold (0.4~0.5 bar), the isotherm exhibits a sharp upward inflection, followed by a steep and continuous rise in capacity, with the total uptake reaching approximately 1.2 mmol/g at 1.0 bar. Subsequently, in the desorption branch of C_2_H_4_, the isotherm forms an extraordinarily broad desorption hysteresis loop. A precipitous desorption occurs only when the system pressure is reduced to an extremely low level (<0.1 bar). In contrast, the interactions of C_2_H_6_ are completely insufficient to drive the flexible phase transition of the framework. Its adsorption branch merely displays a gradual, low-capacity uptake restricted to the intrinsic pores (yielding only about 0.5 mmol/g at 1.0 bar). Furthermore, the desorption branch almost perfectly overlaps with the adsorption branch, exhibiting a high degree of thermodynamic reversibility without discernible hysteresis.

The single-component adsorption isotherms of C_2_H_4_ and C_2_H_6_ on ZnPaDa at 323 K entirely lose the pronounced step-like and hysteretic features observed at lower temperatures. In the adsorption branch of C_2_H_4_, the isotherm exhibits a rapid initial increase in the low-pressure region before gradually leveling off. No distinct upward inflection or step-like surge is observed across the entire 0–1.0 bar range, culminating in a maximum uptake of only ~0.55 mmol/g at 1.0 bar. Correspondingly, its desorption branch almost perfectly traces the adsorption path, demonstrating a high degree of reversibility with no hysteresis loop. Similarly, the adsorption branch of C_2_H_6_ displays a smooth, continuous rise devoid of any abrupt changes, resulting in an even lower uptake of ~0.4 mmol/g at 1.0 bar. The desorption branch of C_2_H_6_ also directly overlaps with the adsorption curve, exhibiting a fully reversible adsorption–desorption profile.

ZnPaDa exhibits a unique guest- and temperature-dependent gate-opening behavior. At 273 K, C_2_H_4_ demonstrates a pronounced gate-opening effect, characterized by a low threshold pressure and an exceptionally broad hysteresis loop. As the temperature rises to 298 K, the gate-opening pressure shifts to a higher value, accompanied by a contraction of the hysteresis loop. Upon further increasing the temperature to 323 K, the gate-opening effect is completely suppressed, and the hysteresis loop vanishes. For C_2_H_6_, a subtle gate-opening effect and a small hysteresis loop are observed at 273 K; however, as the temperature reaches 298 K and 323 K, this gate-opening behavior is no longer evident, and the adsorption profiles transition into characteristic Type-I isotherms.

As a result, temperature has an obvious regulatory effect on the adsorption capacity: at 1 bar of pressure, the adsorption capacity of ZnPaDa for C_2_H_4_ reaches 1.5 mmol/g and for C_2_H_6_ is 0.75 mmol/g with uptake ratio as 2 at 273 K; at 298 K, these values decrease to 1.2 mmol/g and 0.5 mmol/g, respectively and uptake ratio increases to 2.4; at 323 K, they further decrease to 0.55 mmol/g and 0.42 mmol/g and uptake ratio decreases to 1.3. This behavior underscores the potential of ZnPaDa for highly selective C_2_H_4_/C_2_H_6_ separation at 298 K, which is primarily driven by the temperature-tuned, guest-responsive flexible gate-opening mechanism. The ideal adsorption solution theory (IAST) was employed to calculate the C_2_H_4_/C_2_H_6_ (50/50, *v*/*v*) selectivity at 323 K (Figure 3d) of ZnPaDa by fitting the dual-site Langmuir–Freundlich (DSLF) model (Figure S2 and Table S1). At this temperature, both C_2_H_4_ and C_2_H_6_ isotherms exhibit typical Type I behavior without the abrupt S-shaped transition associated with gate-opening, indicating that the framework behaves pseudo-rigidly toward both adsorbates within the evaluated pressure range. This justifies the application of the DSLF model and IAST calculation under these specific conditions. Within the pressure range of 0~1.0 bar, the IAST selectivity is stably maintained at approximately 3–4 and shows only a slight upward trend as the pressure increases, demonstrating good separation potential at 323 K.

To further clarify the molecular recognition mechanism of ZnPaDa for C_2_H_4_ and C_2_H_6_, we calculated the isosteric heat of adsorption (Q_st_, Figure 3e) as a function of gas uptake by fitting the isotherms to the virial equation (Figure S3 and Table S2). The adsorption heat of C_2_H_4_ is higher than that of C_2_H_6_ across the entire uptake range. For example, upon 0.4 mmol/g uptake, the Q_st_ of C_2_H_4_ and C_2_H_6_ are 30 kJ/mol and 23 kJ/mol, respectively. This marked energy difference provides compelling evidence for stronger host-guest interactions between C_2_H_4_ molecules and the ZnPaDa framework. We attribute this enhanced interaction primarily to the distinct hydrogen-bonding characteristics arising from the difference in hybridization between C_2_H_4_ and C_2_H_6_. The sp2-hybridized C–H bonds in C_2_H_4_ possess higher s-character and are consequently more polarized and acidic than the sp3-hybridized C–H bonds in C_2_H_6_, rendering the vinylic C–H protons of C_2_H_4_ more effective hydrogen-bond donors toward the electronegative oxygen/nitrogen atoms lining the ZnPaDa pore surface. In addition, the π-electron cloud associated with the C=C double bond of C_2_H_4_ might engage in supplementary weak interactions (e.g., C–H···π or dipole-induced interactions) with the framework, which are absent in the saturated, non-polarizable C–C single bond of C_2_H_6_. The synergy of these possible stronger C–H···O(N) hydrogen bonds and the additional π-related interactions might collectively account for the higher Q_st_ observed for C_2_H_4_ relative to C_2_H_6_. This difference in Q_st_ thermodynamically explains why ethylene is more effective than ethane at overcoming the framework’s deformation energy barrier and inducing the gate-opening effect. Figure S4 shows the C_2_H_4_ heat of adsorption (Q_st_) of different materials, used to quantify the interaction strength between the adsorbent and C_2_H_4_ molecules. ZnPaDa’s C_2_H_4_ heat of adsorption is approximately 39.3 kJ/mol, which is in a moderate range among comparative systems: it is higher than weak adsorption materials such as ZU-901 and Zn-ATzPO_4_, ensuring effective adsorption selectivity for C_2_H_4_. It is also lower than high-heat-of-adsorption materials such as Co-gallate and Mg-MOF-74, effectively avoiding high energy consumption during the adsorbent regeneration process and achieving a balance between adsorption selectivity and regeneration energy consumption.

Beyond the thermodynamic evidence provided by Q_st_, we further sought direct structural evidence of this gate-opening phenomenon via ex situ PXRD measurements at 298 K. The time-dependent PXRD data collectively demonstrate that ZnPaDa is a flexible framework undergoing reversible phase transitions in response to guest removal and gas adsorption. As shown in Figure 3f, upon activation, the “Activated ZnPaDa” sample exhibits pronounced structural changes relative to the “As-synthesized ZnPaDa”, including the emergence of a new peak (2θ = 7.6–8.2°), a marked intensity change within 2θ = 10.2–11°, peak splitting within 2θ = 11.8–12.8°, and peak shifts within 2θ = 14.8–15.4° and 17.6–18.4°, confirming that removal of guest solvent molecules induces a phase transition inherent to the framework’s structural flexibility. This transition proves to be reversible: upon exposure of the activated sample to air, the “ZnPaDa@Air-6 min” and “ZnPaDa@Air-12 min” samples show a progressive reversion toward the original as-synthesized phase. Specifically, the newly emerged peak (2θ = 7.6–8.2°) disappears entirely, the peak intensity within 2θ = 10.2–11° declines progressively and eventually approaches that of the as-synthesized sample after 12 min, and the split peak within 2θ = 11.8–12.8° becomes progressively less resolved before merging back into a single peak, closely resembling the as-synthesized state. These observations confirm that the structural changes accompanying desorption and adsorption are dynamically reversible, further corroborating the flexible nature of the framework. Notably, when the activated sample is directly exposed to ethylene (“ZnPaDa@C_2_H_4_”), the PXRD pattern undergoes essentially the same set of transformations observed upon air exposure: the newly emerged peak (2θ = 7.6–8.2°) disappears, the peak within 2θ = 10.2–11° decreases slightly, and the splitting within 2θ = 11.8–12.8° is reduced, providing direct, unambiguous evidence that gas adsorption itself triggers the same phase transition, and that the framework actively reconfigures its structure to accommodate the incoming gas molecules.

### 2.4. Discussion on Mixed-Gas Breakthrough Behavior

**Figure 4:**
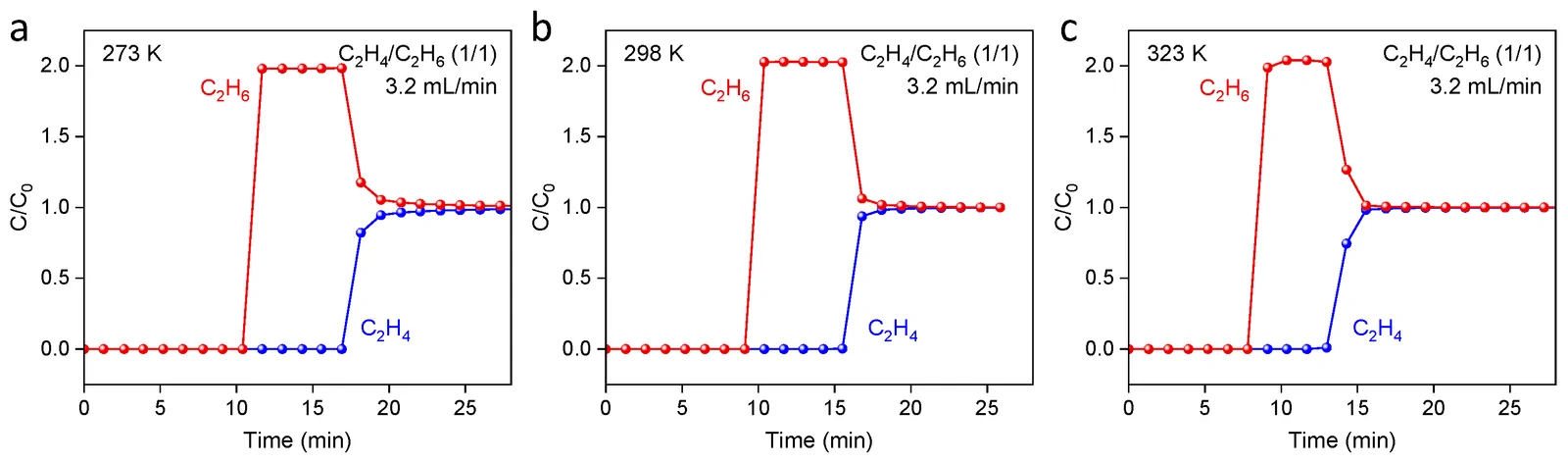
Mixed-gas C_2_H_4_/C_2_H_6_ (1:1) fixed-bed breakthrough experiments of ZnPaDa at 273 K (**a**), 298 K (**b**) and 323 K (**c**).

The breakthrough curve tests for a C_2_H_4_/C_2_H_6_ (1:1) binary gas mixture on ZnPaDa were conducted at 273 K, 298 K and 323 K, with test conditions maintained at 1 bar of pressure, a flow rate of 3.2 mL/min, and the same column specification (4.6 × 150 mm), as shown in Figure 4. It can be seen from the figure that ZnPaDa exhibits significant preferential adsorption for C_2_H_4_ achieving efficient separation of the two gases. At both temperatures, C_2_H_6_ breakthrough occurs prior to C_2_H_4_, reflecting the material’s strong adsorption affinity and rapid mass transfer characteristics within its pores. Even when the temperature is increased from 273 K to 298 K, ZnPaDa maintains a high adsorption capacity for C_2_H_4_ and excellent C_2_H_4_/C_2_H_6_ separation selectivity without significant attenuation, fully demonstrating the material’s exceptional temperature adaptability and application potential in the field of olefin/alkane separation.

To rigorously demonstrate the potential of ZnPaDa for the dynamic separation of C_2_H_4_/C_2_H_6_ mixtures, we further conducted breakthrough experiments at various temperatures (Figure 4). Under conditions of 1 bar and a flow rate of 3.2 mL/min, ZnPaDa exhibits a significant retention capacity for C_2_H_4_. Quantitative experimental data show that at 273 K, the breakthrough time for C_2_H_6_ is ~11.0 min, whereas the breakthrough of C_2_H_4_ is delayed until ~18.0 min, resulting in a separation window of 7.0 min and a dynamic separation ratio of 4.29. When the temperature is increased to 298 K, although the breakthrough time for C_2_H_6_ shifts earlier to ~9.0 min, the breakthrough time for C_2_H_4_ remains at ~16.5 min, expanding the separation window to 7.5 min and reaching a peak separation ratio of 5.38. This indicates that at this temperature, the thermodynamic gate-opening effect and kinetic adsorption achieve an optimal synergistic state. As the temperature is further increased to 323 K, the breakthrough times for C_2_H_6_ and C_2_H_4_ shorten to ~8.0 min and ~14.0 min, respectively, due to the reduced adsorption affinity of the framework for C_2_H_4_ at higher temperatures, causing the separation window to narrow to 6.0 min and the separation ratio to decline to 5.04. In summary, the breakthrough experimental results not only verify the feasibility of ZnPaDa for efficient C_2_H_4_/C_2_H_6_ separation at ambient temperature but also identify 298 K as the optimal operating temperature window for achieving maximum separation efficiency.

Overall, ZnPaDa exhibits a balanced and excellent comprehensive performance in C_2_H_4_/C_2_H_6_ separation: it possesses a considerable adsorption capacity, a moderate heat of adsorption (low regeneration energy consumption), and high separation selectivity. This overcomes the bottleneck of traditional materials where “high selectivity is accompanied by high energy consumption, and high capacity is accompanied by low selectivity,” providing a potential new MOF adsorbent for industrial separation.

## 3. Materials and Methods

### 3.1. Reagents and Instruments

All chemical reagents and gases used in the experiments were purchased from commercial sources and used as received. Zn(NO_3_)_2_·6H_2_O (99.9%) and 3,5-diamino-1,2,4-triazole (98%) were obtained from Shanghai Macklin Biochemical Co., Ltd. (Shanghai, China) Pyromellitic acid (C_10_H_6_O_8_, 98%) and acetonitrile (CH_3_CN, AR, ≥99.5%) were purchased from Sinopharm Chemical Reagent Co., Ltd. (Shanghai, China). High-purity gases, including N_2_ (99.999%), He (99.999%), C_2_H_4_ (99.999%), C_2_H_6_ (99.999%), and a C_2_H_4_/C_2_H_6_ mixture (50%/50%), were supplied by Air Liquide (Tianjin) Co., Ltd. (Tianjin, China).

X-ray diffraction (XRD) was conducted using a Bruker D2 diffractometer (Bruker, Berlin, Germany). Scanning electron microscopy (SEM) images were obtained using a Sigma300 microscope (Carl Zeiss, Jena, Germany). Thermogravimetric analysis (TGA) was performed on a HENVEN HCT-04 thermal analyzer (Beijing Henven Scientific Instrument Factory, Beijing, China).

Gas adsorption analysis was carried out using a BSD-VVS intelligent gravimetric adsorption analyzer (BSD Instrument, Beijing, China). Gas chromatography (GC) analysis was performed using a GC460 gas chromatograph (Shanghai Huishi Instrument Equipment Co., Ltd., Shanghai, China). Specific surface area and pore size distribution were measured using a BSD-660M (BSD Instrument, Beijing, China).

### 3.2. Preparation of ZnPaDa

ZnPaDa was synthesized following a previously reported procedure with minor modifications [41]. Zn(NO_3_)_2_·6H_2_O (1.787 g, 6 mmol) and 3,5-diamino-1,2,4-triazole (0.594 g, 6 mmol) were dissolved in deionized water (30 mL), and pyromellitic acid (1.524 g, 6 mmol) was separately dispersed in acetonitrile (30 mL). The two solutions were combined in a hydrothermal reaction kettle and heated at 433 K for 60 h to yield a white powder. The product was washed with methanol, subjected to solvent exchange in methanol for 2 days, and dried under vacuum at 353 K for 12 h to obtain activated ZnPaDa. Full synthetic details are provided in the Supporting Information.

### 3.3. Characterization of ZnPaDa

#### 3.3.1. Powder X-Ray Diffraction

Powder X-ray diffraction (PXRD) patterns were collected using a Bruker D2 Phaser diffractometer (Bruker, Berlin, Germany) equipped with Cu Kα radiation (*λ* = 1.540598 Å) at 30 kV and 10 mA. Data were scanned over a 2θ range of 5–50° with a step size of 0.16°/s.

To investigate the structural flexibility of ZnPaDa, PXRD patterns at 298 K of the as-synthesized sample and the activated sample (obtained by degassing at 353 K for 12 h) were compared. To further examine the reversibility of this structural transformation, the activated sample was exposed to ambient air, and PXRD patterns were subsequently recorded after 6 min (“ZnPaDa@Air-6 min”) and 12 min (“ZnPaDa@Air-12 min”) to monitor the structural evolution accompanying gas adsorption from air.

In addition, to directly probe the gas-induced structural transition, the activated sample was loaded with C_2_H_4_ at 1 bar and 298 K for 0.5 h, and then the sample was quickly loaded in the Bruker D2 Phaser diffractometer, and resulting PXRD pattern (“ZnPaDa@C_2_H_4_”) was collected under the same instrumental conditions described above for comparison with the as-synthesized, activated, and air-exposed samples.

#### 3.3.2. Thermogravimetric Analysis

Thermogravimetric Analysis (TGA) was performed using an HENVEN HCT-04 thermal analyzer (Beijing Henven Scientific Instrument Factory). Approximately 10–15 mg of the sample was placed in a thermogravimetric crucible. The test was conducted under a compressed dry air (CDA) atmosphere over a temperature range of 25–800 °C at a heating rate of 5 °C/min to obtain the sample’s thermal weight loss curve.

#### 3.3.3. Scanning Electron Microscopy

Scanning Electron Microscopy (SEM) images were captured using a Sigma300 scanning electron microscope (Carl Zeiss, Jena, Germany) to characterize the microstructure of the materials. The operating voltage was 10 kV. Prior to testing, a small amount of the sample was evenly coated onto a conductive adhesive surface, followed by gold sputtering to enhance conductivity and ensure imaging quality. After the instrument was preheated and stable, the gold-coated sample was placed into the sample chamber under vacuum. Parameters were adjusted to preview the sample at low magnification; target areas were then selected for high-magnification imaging.

#### 3.3.4. Specific Surface Area and Pore Structure

The specific surface area, pore volume, and pore size distribution were determined using a BSD-660M fully automatic specific surface and micropore analyzer (BSD Instrument, Beijing, China) by measuring CO_2_ adsorption–desorption isotherms. Before testing, approximately 100 mg of the sample was degassed in a sample tube at 373 K under vacuum for 6 h to thoroughly remove adsorbed solvents and impurities. During the test, the sample tube was immersed in a Dewar flask filled with liquid nitrogen to control the temperature, and CO_2_ was used as the adsorbate at 195 K. After the instrument was set to the micropore analysis mode, data were collected. The specific surface area was calculated using the Brunauer–Emmett–Teller (BET) equation, and the pore size distribution was analyzed using the Horvath–Kawazoe (HK) model.

#### 3.3.5. Fourier Transform Infrared Spectroscopy

Fourier Transform Infrared (FTIR) spectroscopy was performed using a Vector33 Fourier transform infrared spectrometer (Bruker, Berlin, Germany). The samples were prepared by uniformly grinding and mixing the MOFs with KBr powder and pressing the mixture into thin pellets. Since different functional groups exhibit distinct absorption frequencies for infrared light, they show characteristic peaks at different positions in the spectra. By analyzing the position, intensity, and width of these characteristic peaks, the types of functional groups in the MOFs were qualitatively identified.

#### 3.3.6. X-Ray Photoelectron Spectroscopy

X-ray photoelectron spectroscopy (XPS) was performed using a Thermo Scientific K-Alpha spectrometer. For sample preparation, the target material was cleaned with analytical grade anhydrous ethanol and dried at 333 K under a vacuum of 10^−3^ Pa for 2 h to remove moisture. The powder was then spread evenly onto conductive adhesive, mounted onto the sample holder, and introduced into the analysis chamber once the required vacuum was reached. The instrument was preheated and calibrated for 30 min prior to the experiment. A monochromatic Al Kα X-ray source (*hν* = 1486.6 eV) was used with a gun power of 150 W and a spot size of 400 μm. The sample chamber was maintained at a high vacuum of at least 10^−9^ Pa to improve the signal-to-noise ratio. Full-spectrum analysis was conducted over 0–1350 eV (step size 1 eV, 10 scans) to determine elemental composition, followed by high-resolution scans for specific elements (step size 0.05 eV, 20 scans) to analyze chemical states. A charge neutralizer was used during the test to compensate for charging effects. Data were processed using XPS Peak software Thermo Avantage v5.948.

### 3.4. Single-Component Adsorption Experiment

The adsorption isotherms of C_2_H_4_ and C_2_H_6_ on the target sample were measured at 273 K, 298 K, and 323 K using a BSD-VVS intelligent gravimetric adsorption analyzer (BSD Instrument, Beijing). Before each adsorption test, the sample underwent degassing pretreatment: approximately 100 mg of the sample was weighed and heated at 353 K under vacuum for 4–6 h to thoroughly remove impurities and residual gases adsorbed within the pores. The pressure range for the adsorption tests was set from 0 to 100 kPa, and the test temperature was precisely controlled by placing the sample tube in a Dewar flask connected to a circulating water bath.

### 3.5. Mixed-Gas Fixed-Bed Breakthrough Experiment

The adsorption breakthrough curves for the C_2_H_4_/C_2_H_6_ (1:1, *v*/*v*) binary gas mixture on the ZnPaDa material were determined using a laboratory-built fixed-bed adsorption breakthrough apparatus, the schematic diagram of which is shown in Figure S5. Prior to testing, the powder sample was degassed and activated in a vacuum oven at 353 K for 12 h. After cooling, the sample was packed into a stainless steel adsorption column with dimensions of Φ4.6 × 150 mm. The column was then connected to a nitrogen line and placed in an oven at 373 K for 12 h to purge residual impurities and other substances from the pores. Upon completion of the activation process, the activated stainless steel adsorption column was connected to the breakthrough testing apparatus (which had been pre-purged with nitrogen to remove impurity gases from the lines). Subsequently, the flow rate of the C_2_H_4_/C_2_H_6_ (1:1, *v*/*v*) mixed gas was regulated by mass flow controllers to 3.2 mL/min, respectively, with the test temperature set at 298 K. The composition of the gas at the column outlet was analyzed using a gas chromatograph. Once the composition of the mixed gas detected by the chromatograph remained constant, the gas valves and the thermostatic device were shut down. By measuring the mixed gas breakthrough curves, the actual adsorption and separation characteristics of the material can be further investigated.

## 4. Conclusions

In this work, we have demonstrated that the flexible ZnPaDa framework achieves high-performance C_2_H_4_/C_2_H_6_ separation through a unique, temperature-responsive adsorption mechanism. The material exhibits a remarkable evolution in its adsorption isotherms as temperature increases, transitioning from a state at lower temperatures where both C_2_H_4_ and C_2_H_6_ induce a gate-opening effect, to an intermediate regime where this effect is selectively triggered only by C_2_H_4_, and finally to a state at higher temperatures where the gate-opening behavior remains absent for both gases. This guest- and temperature-dependent behavior is driven by the stronger thermodynamic affinity of C_2_H_4_ for the framework, which allows it to more effectively overcome the energy barrier required for this adsorption transition compared to C_2_H_6_. Notably, 298 K emerges as the optimal operating temperature, as it simultaneously preserves a relatively high ethylene adsorption capacity while reaching the maximum static adsorption ratio, which coincides with the highest dynamic separation selectivity observed in breakthrough experiments. Consequently, the superior performance of ZnPaDa, governed by its tunable response, establishes it as a highly promising candidate for energy-efficient ethylene purification.

## Data Availability

The original contributions presented in this study are included in the article/Supplementary Material. Further inquiries can be directed to the corresponding author.

## Supplementary Materials

The following supporting information can be downloaded at: https://www.mdpi.com/article/10.3390/molecules31162875/s1, Figure S1: Pore size distributions (calculated using the Horvath–Kawazoe method) of ZnPaDa; Figure S2: Adsorption isotherms of C_2_H_4_ and C_2_H_6_ on ZnPaDa at 323 K fitted with the DSLF model; Figure S3: Global virial fitting curves for C_2_H_4_ (a) and C_2_H_6_ (b) adsorption on ZnPaDa at 273 K, 298 K and 323 K; Figure S4: Qst comparison of a series MOFs; Figure S5: Schematic diagram of the C_2_H_4_/C_2_H_6_ mixed gas breakthrough experiments; Table S1: Fitting parameters of the DSLF model for C_2_H_4_ and C_2_H_6_ adsorption on ZnPaDa at 323 K; Table S2: Virial fitting parameters of C_2_H_4_ and C_2_H_6_ adsorption on ZnPaDa.

## References

[1] Li J.R., Sculley J., Zhou H.C. (2012). Metal-Organic Frameworks for Separations. Chem. Rev..

[2] Herm Z.R., Bloch E.D., Long J.R. (2013). Hydrocarbon Separations in Metal–Organic Frameworks. Chem. Mater..

[3] Sholl D.S., Lively R.P. (2016). Seven chemical separations to change the world. Nature.

[4] Wang H., Luo D., Velasco E., Yu L., Li J. (2021). Separation of alkane and alkene mixtures by metal–organic frameworks. J. Mater. Chem. A.

[5] Xie W.P., Yang L.Z., Zhang J., Zhao X.B. (2023). The Adsorptive Separation of Ethylene from C_2_ Hydrocarbons by Metal-Organic Frameworks. Chem.-Eur. J..

[6] Liao P.Q., Zhang W.X., Zhang J.P., Chen X.M. (2015). Efficient purification of ethene by an ethane-trapping metal-organic framework. Nat. Commun..

[7] Ren T., Patel M., Blok K. (2006). Olefins from conventional and heavy feedstocks: Energy use in steam cracking and alternative processes. Energy.

[8] Mofarahi M., Salehi S.M. (2013). Pure and binary adsorption isotherms of ethylene and ethane on zeolite 5A. Adsorption.

[9] Seabra R., Martins V.F.D., Ribeiro A.M., Rodrigues A.E., Ferreira A.P. (2021). Ethylene/ethane separation by gas-phase SMB in binderfree zeolite 13X monoliths. Chem. Eng. Sci..

[10] Huang B.L., Zhu L., Du Z.L., Anjum A.W., Li X.X., Li X.N., Miao G., Xiao J., Du S.J. (2024). Highly efficient ethylene/ethane separation via molecular sieving using chitosan-derived ultramicroporous carbon. Sep. Purif. Technol..

[11] Liang W.W., Zhang Y.F., Wang X.J., Wu Y., Zhou X., Xiao J., Li Y.W., Wang H.H., Li Z. (2017). Asphal-derived high surface area activated porous carbons for the effective adsorption separation of ethane and ethylene. Chem. Eng. Sci..

[12] Li Y.T., Li W.L., Zhang L.P., Xu L., Jiang Y., Wang S.M., Li X.X., Yang Q.Y., Guan Q.Q., Zaworotko M.J. (2026). Stimulus-Induced Microflexible Nanopores in Zinc-Based Coordination Network Enable Four Distinct Adsorption Mechanisms for Efficient Light Hydrocarbon Separations. J. Am. Chem. Soc..

[13] Bloch E.D., Queen W.L., Krishna R., Zadrozny J.M., Brown C.M., Long J.R. (2012). Hydrocarbon Separations in a Metal-Organic Framework with Open Iron(II) Coordination Sites. Science.

[14] Lin R.-B., Li L., Zhou H.-L., Wu H., He C., Li S., Krishna R., Li J., Zhou W., Chen B. (2018). Molecular sieving of ethylene from ethane using a rigid metal–organic framework. Nat. Mater..

[15] Bao Z., Wang J., Zhang Z., Xing H., Yang Q., Yang Y., Wu H., Krishna R., Zhou W., Chen B. (2018). Molecular Sieving of Ethane from Ethylene Through the Molecular Cross-Section Size Differentiation in Gallate-Based Metal–Organic Frameworks. Angew. Chem..

[16] Martins V.F.D., Ribeiro A.M., Ferreira A., Lee U.H., Hwang Y.K., Chang J.-S., Loureiro J.M., Rodrigues A.E. (2015). Ethane/ethylene separation on a copper benzene-1,3,5-tricarboxylate MOF. Sep. Purif. Technol..

[17] Li L., Lin R.-B., Krishna R., Li H., Xiang S., Wu H., Li J., Zhou W., Chen B. (2018). Ethane/ethylene separation in a metal-organic framework with iron-peroxo sites. Science.

[18] Chen Y., Qiao Z., Wu H., Lv D., Shi R., Xia Q., Zhou J., Li Z. (2018). An ethane-trapping MOF PCN-250 for highly selective adsorption of ethane over ethylene. Chem. Eng. Sci..

[19] Li J.-R., Kuppler R.J., Zhou H.-C. (2009). Selective gas adsorption and separation in metal–organic frameworks. Chem. Soc. Rev..

[20] Wu H.H., Gong Q.H., Olson D.H., Li J. (2012). Commensurate Adsorption of Hydrocarbons and Alcohols in Microporous Metal Organic Frameworks. Chem. Rev..

[21] Zhou H.C., Long J.R., Yaghi O.M. (2012). Introduction to Metal-Organic Frameworks. Chem. Rev..

[22] Silva P., Vilela S.M.F., Tomé J.P.C., Almeida Paz F.A. (2015). Multifunctional metal–organic frameworks: From academia to industrial applications. Chem. Soc. Rev..

[23] Schoedel A., Li M., Li D., O’Keeffe M., Yaghi O.M. (2016). Structures of Metal-Organic Frameworks with Rod Secondary Building Units. Chem. Rev..

[24] He Y., Krishna R., Chen B. (2012). Metal–organic frameworks with potential for energy-efficient adsorptive separation of light hydrocarbons. Energy Environ. Sci..

[25] Chang G., Huang M., Su Y., Xing H., Su B., Zhang Z., Yang Q., Yang Y., Ren Q., Bao Z. (2015). Immobilization of Ag(i) into a metal–organic framework with –SO_3_H sites for highly selective olefin–paraffin separation at room temperature. Chem. Commun..

[26] Li B., Zhang Y., Krishna R., Yao K., Han Y., Wu Z., Ma D., Shi Z., Pham T., Space B. (2014). Introduction of π-Complexation into Porous Aromatic Framework for Highly Selective Adsorption of Ethylene over Ethane. J. Am. Chem. Soc..

[27] Ma Y.X., Yu C., Yang L.F., You R.M., Bo Y.W., Gong Q.H., Xing H.B., Cui X.L. (2024). Boosting kinetic separation of ethylene and ethane on microporous materials via crystal size control. Chin. J. Chem. Eng..

[28] Pires J., Pinto M.L., Saini V.K. (2014). Ethane Selective IRMOF-8 and Its Significance in Ethane-Ethylene Separation by Adsorption. ACS Appl. Mater. Interfaces.

[29] Zhang L., Yu B., Wang M., Chen Y., Wang Y., Sun L.B., Zhang Y.B., Zhang Z.J., Li J.P., Li L.B. (2025). Ethane Triggered Gate-Opening in a Flexible-Robust Metal-Organic Framework for Ultra-High Purity Ethylene Purification. Angew. Chem. Int. Ed..

[30] Arami-Niya A., Birkett G., Zhu Z.H., Rufford T.E. (2017). Gate opening effect of zeolitic imidazolate framework ZIF-7 for adsorption of CH_4_ and CO_2_ from N_2_. J. Mater. Chem. A.

[31] Chen Y., Qiao Z., Lv D., Duan C., Sun X., Wu H., Shi R., Xia Q., Li Z. (2017). Efficient adsorptive separation of C_3_H_6_ over C_3_H_8_ on flexible and thermoresponsive CPL-1. Chem. Eng. J..

[32] Kim H.C., Huh S., Lee D.N., Kim Y. (2018). Selective carbon dioxide sorption by a new breathing three-dimensional Zn-MOF with Lewis basic nitrogen-rich channels. Dalton Trans..

[33] Dong Q.B., Zhang X., Liu S., Lin R.B., Guo Y.N., Ma Y.S., Yonezu A., Krishna R., Liu G.P., Duan J.G. (2020). Tuning Gate-Opening of a Flexible Metal-Organic Framework for Ternary Gas Sieving Separation. Angew. Chem. Int. Ed..

[34] Acharya S.R., Elias A., Tan K., Jensen S., Lin R.-B., Chen B., Gross M.D., Thonhauser T. (2022). Identifying the Gate-Opening Mechanism in the Flexible Metal–Organic Framework UTSA-300. Inorg. Chem..

[35] Nikolayenko V.I., Castell D.C., Sensharma D., Shivanna M., Loots L., Forrest K.A., Solanilla-Salinas C.J., Otake K.I., Kitagawa S., Barbour L.J. (2023). Reversible transformations between the non-porous phases of a flexible coordination network enabled by transient porosity. Nat. Chem..

[36] Wang S.M., Shivanna M., Zheng S.T., Pham T., Forrest K.A., Yang Q.Y., Guan Q.Q., Space B., Kitagawa S., Zaworotko M.J. (2024). Ethane/Ethylene Separations in Flexible Diamondoid Coordination Networks via an Ethane-Induced Gate-Opening Mechanism. J. Am. Chem. Soc..

[37] Zhang W.J., Zou S.X., Zhou Y.Z., Ji Z.Y., Li H.B., Zhen G.L., Chen C., Song D.H., Wu M.Y. (2024). Flexible Microporous Framework for One-Step Acquisition of Ethylene from Ternary C_2_ Hydrocarbons. Inorg. Chem..

[38] Wang K., Jiang Y., Zhang H., Jia S.J., Wang Q., Cui P., Alshahrani T., Ma S.Q. (2025). Engineering the Microporous Environment of Flexible Metal-Organic Frameworks with Bifunctionality for Promoting the Separation of Ethylene from a Ternary Mixture. Angew. Chem. Int. Ed..

[39] Mukherjee D., Pal S.C., Wang J.X., Bon V., Chand S., Kaskel S., Li B., Volkmer D., Das M.C. (2025). Two-in-One Flexible Metal-Organic Framework: One-Step C_2_H_4_ Purification via Inverse C_2_H_6_-C_2_H_4_ and C_2_H_2_-CO_2_ Separations. J. Am. Chem. Soc..

[40] Zhang X.W., He H., Gan Y.W., Wang Y., Huang N.Y., Liao P.Q., Zhang J.P., Chen X.M. (2024). High-Pressure Molecular Sieving of High-Humidity C_2_H_4_/C_2_H_6_ Mixture by a Hydrophobic Flexible Metal-Organic Framework. Angew. Chem. Int. Ed..

[41] Cui P.L., Li X.H. (2015). Construction of two new Zn(II)-guanazole frameworks varying organic carboxylate ligands. J. Mol. Struct..

