# Supplementary material for: Efficient Ethylene/Ethane Separation Achieved by a Temperature-Responsive Flexible MOF
Source: Molecules. 2026 Aug 18;31(16):2875. doi: 10.3390/molecules31162875 (PMC13515805; doi:10.3390/molecules31162875)
Supplement: Supplementary file 1 [file molecules-31-02875-s001.zip › molecules-4480370-supplementary.pdf]

Supporting Information

Efficient Ethylene/Ethane Separation Achieved by a Temperature-Responsive Flexible MOF

Sheng Liu , Xuefu Tian , Yutong Zou , Yanxi Li, Yawen Bo, Chuanqi Wu, Kebin Chi, Dejun Shi and Qihan Gong*

Fundamental Science & Advanced Technology Lab, PetroChina Petrochemical Research Institute, Beijing 102200, China

* Corresponding authors.

E-mail addresses: gongqihan@petrochina.com.cn (Q. Gong).

Ideal adsorbed solution theory (IAST) selectivity calculations

To evaluate the separation selectivity, the dual-site Langmuir–Freundlich (DSLF) model was used to fit the single-component adsorption isotherms of C_2H_4 and C_2H_6. The adsorption isotherms and gas selectivity for C_2H_4/C_2H_6 (50/50, v/v) at 273 K were calculated using the ideal adsorbed solution theory (IAST).

The DSLF model is listed below:

$$N = N_1^{\max} \times \frac{b_1 P^{c_1}}{1 + b_1 P^{c_1}} + N_2^{\max} \times \frac{b_2 P^{c_2}}{1 + b_2 P^{c_2}} \tag{S1}$$

where P (bar) is the pressure of the bulk gas at equilibrium with the adsorbed phase, N (mmol g^{-1}) is the adsorbed amount per mass of adsorbent, $N_1^{\max}$ and $N_2^{\max}$ (mmol g^{-1}) are the saturated capacities of site 1 and site 2, b_1 and b_2 (bar$^{-c_1}$ and bar$^{-c_2}$) are the affinity coefficients of site 1 and site 2, and c_1 and c_2 represent the deviations from an ideal homogeneous surface.

The adsorption selectivity of C_2H_4 (component 1) over C_2H_6 (component 2) based on IAST for the gas-mixture of C_2H_4/C_2H_6 (50/50, v/v) is defined by

$$S_{\text{ads}} = \frac{q_1 / q_2}{p_1 / p_2} \tag{S2}$$

where q_1 and q_2 are the equilibrated molar loadings of component 1 and 2 in the adsorbed phase with the bulk gas phase at partial pressures of p_1 and p_2, respectively.

Calculations of isosteric enthalpy of adsorption (Q_{st})

The adsorption data for C_2H_4 and C_2H_6 at temperatures of 273 K, 283 K, and 298 K were used to calculate the isosteric enthalpy of adsorption (Q_{st}) and evaluate the binding strength between the adsorbent and adsorbate. The virial equation was used to fit the

adsorption curves:

$$\ln p = \ln q + \frac{1}{T}\sum_{i=0}^{m} a_i q^i + \sum_{i=0}^{n} b_i q^i \tag{S3}$$

where p (P/P_0) is the relative pressure, T (K) is the experimental temperature, q (mmol g^{-1}) is the gas uptake, a_i and b_i are virial coefficients, and m and n are the numbers of terms required to describe the isotherm adequately. The values of the virial coefficients a_0 through a_m were then used to calculate Q_{st} using the following expression:

$$Q_{st} = -R\sum_{i=0}^{m} a_i q^i \tag{S4}$$

where R is the universal gas constant.

Detailed Synthetic Procedure of ZnPaDa

1.787 g (6 mmol) of zinc nitrate ($Zn(NO_3)_2 \cdot 6H_2O$) and 0.594 g (6 mmol) of 3,5-diamino-1,2,4-triazole ($C_2H_5N_5$) were weighed into a 100 mL beaker, and 30 mL of deionized water was added. The mixture was then placed in an ultrasonic cleaner and sonicated for 30 min to ensure full dispersion into a homogeneous solution. Separately, 1.524 g (6 mmol) of pyromellitic acid ($C_{10}H_6O_8$) was weighed and added to a 100 mL hydrothermal reaction kettle, followed by the addition of 30 mL of acetonitrile (C_2H_3N). The reaction kettle was then placed in an ultrasonic cleaner to fully dissolve the solution. Finally, the mixed solution containing zinc nitrate and 3,5-diamino-1,2,4-triazole was transferred into the reaction kettle using a pipette and subjected to further ultrasonic treatment. After sealing the kettle, it was placed in an oven at 433 K for 60 h. Upon completion of the reaction, the kettle was removed and allowed to cool to room temperature. The product was then transferred to a centrifuge tube, washed three times with methanol using a centrifuge at 4000 rpm for 3 min each time, yielding a white powdery product. Subsequently, the collected product was soaked in methanol for

solvent exchange for two days, with the methanol being refreshed twice daily. Finally, the ZnPaDa product was collected in a crucible and dried in a vacuum oven at 353 K for 12 h to obtain the activated sample.

Supplementary Figures

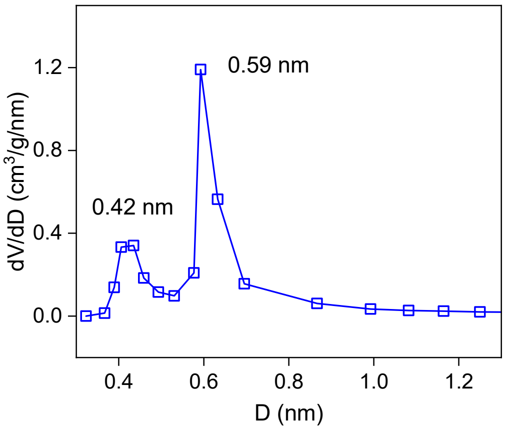

Figure S1. Pore size distributions (calculated using the Horvath–Kawazoe method) of ZnPaDa.

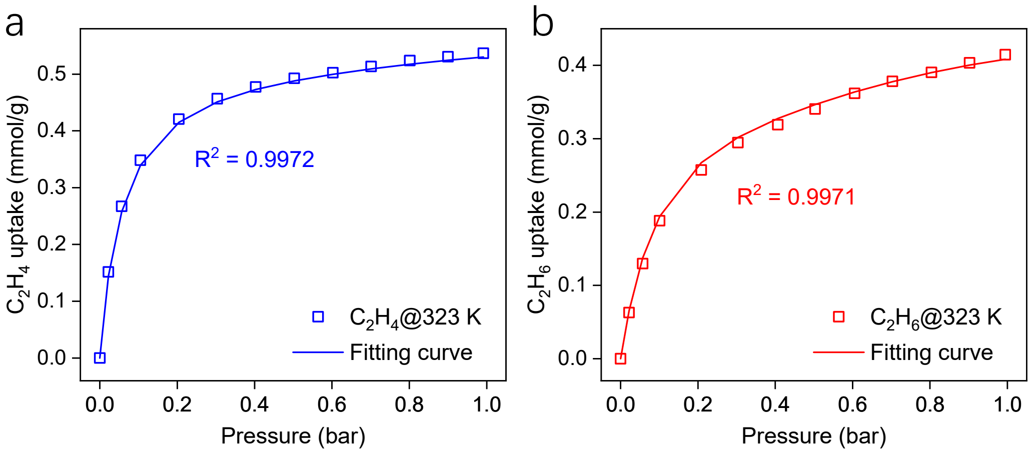

Figure S2. Adsorption isotherms of C_2H_4 and C_2H_6 on ZnPaDa at 323 K fitted with the DSLF model.

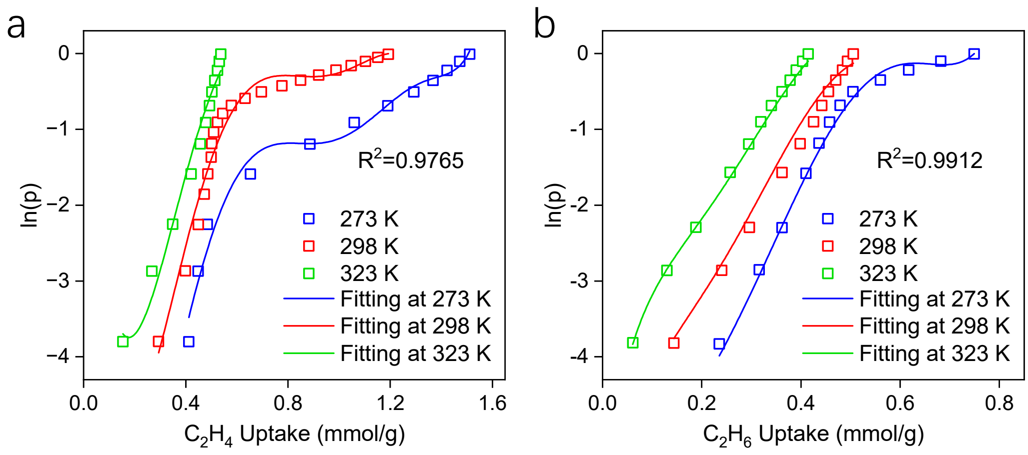

Figure S3. Global virial fitting curves for C_2H_4 (a) and C_2H_6 (b) adsorption on ZnPaDa at 273 K, 298 K and 323 K.

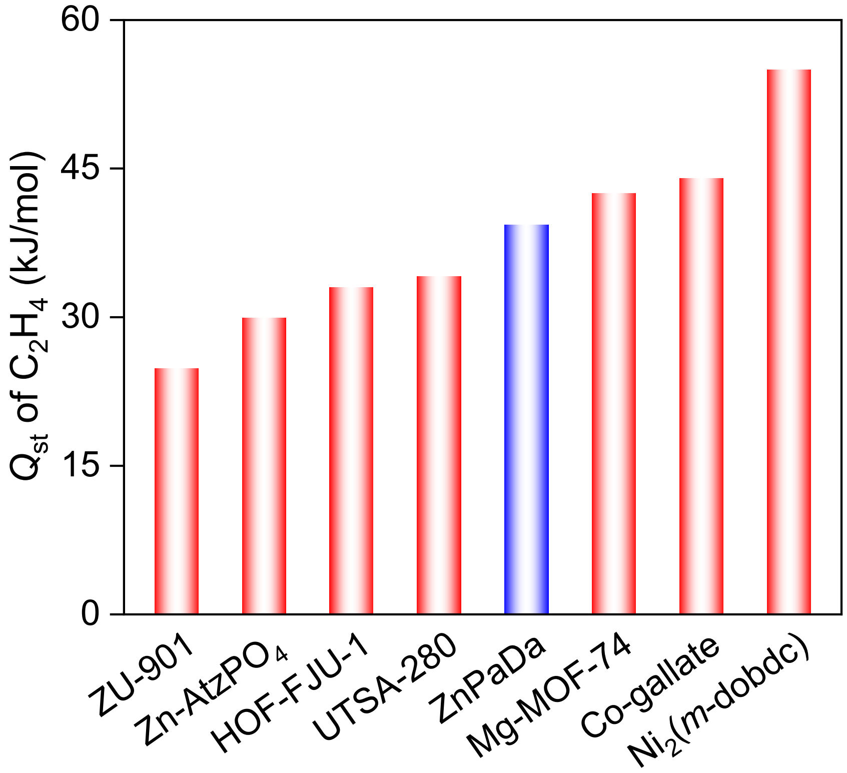

Figure S4. Q_{st} comparison of a series MOFs.

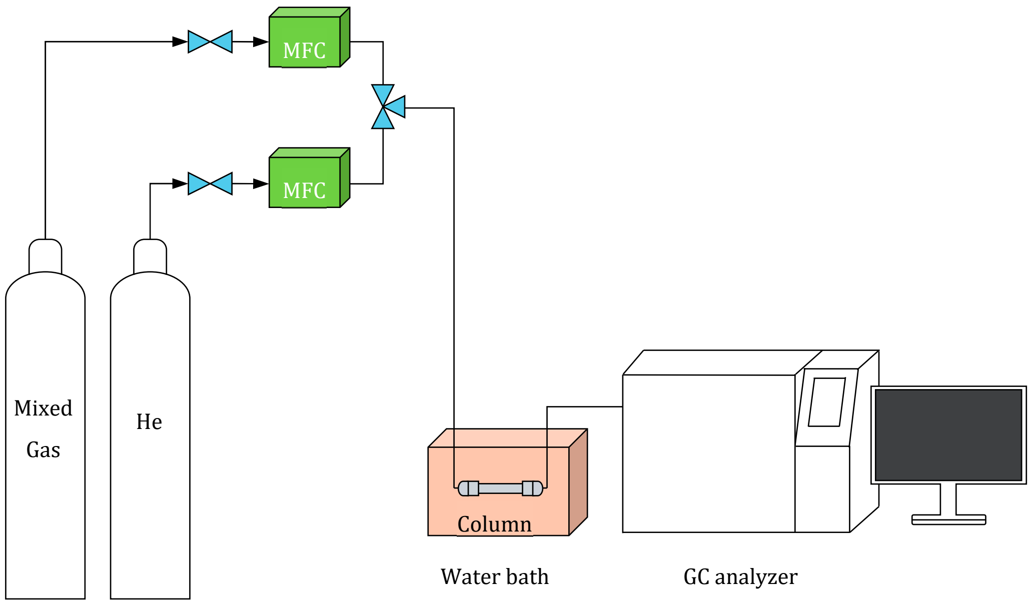

Figure S5. Schematic diagram of the C_2H_4/C_2H_6 mixed gas breakthrough experiments.

Supplementary tables

Table S1. Fitting parameters of the DSLF model for C_2H_4 and C_2H_6 adsorption on ZnPaDa at 323 K.

Fitting parameter	323 K	
	C_2H_4	C_2H_6
N_1^{max} (mmol g^{-1})	0.0364	0.0693
b_1 (bar$^{-c_1}$)	0.9237	2.0177
c_1	2.7615	2.896
N_2^{max} (mmol g^{-1})	0.5483	0.398
b_2 (bar$^{-c_2}$)	14.662	10.2227
c_2	0.9766	1.0357
R^2	0.9972	0.9971

Table S2. Virial fitting parameters of C_2H_4 and C_2H_6 adsorption on ZnPaDa.

Fitting parameter	ZnPaDa	
	C_2H_4	C_2H_6
a0	-4747.639	-4222.63
a1	2593.189	66.08852
a2	1265.416	6004.931
a3	-740.1372	6067.733
a4	-1535.636	4338.2
a5	-976.5128	2694.159
a6	207.7969	1503.631
a7	975.6228	727.7787
a8	553.4596	261.6005
a9	-699.0252	7.532267
a10	273.6972	-111.392
b0	18.53658	11.50348
b1	-80.36824	13.49532
b2	295.8425	-124.061
b3	-502.8855	415.8402
b4	395.9914	-708.424
b5	-117.8734	345.2259
R^2	0.9765	0.9912
